# Experimental models of antibiotic exposure and atopic disease

**DOI:** 10.3389/falgy.2024.1455438

**Published:** 2024-10-25

**Authors:** Katherine Donald, B. Brett Finlay

**Affiliations:** ^1^ Michael Smith Laboratories, University of British Columbia, Vancouver, BC, Canada; ^2^ Department of Microbiology and Immunology, University of British Columbia, Vancouver, BC, Canada; ^3^ Department of Biochemistry and Molecular Biology, University of British Columbia, Vancouver, BC, Canada

**Keywords:** allergies, atopy & microbiome, gut microbiota, antibiotics – immune effect, animal models, cell culture models

## Abstract

In addition to numerous clinical studies, research using experimental models have contributed extensive evidence to the link between antibiotic exposure and atopic disease. A number of mouse models of allergy have been developed and used to uncover the specific effects of various microbiota members and perturbations on allergy development. Studies in mice that lack microbes entirely have also demonstrated the various components of the immune system that require microbial exposure. The importance of the early-life period and the mechanisms by which atopy “protective” species identified in human cohorts promote immune development have been elucidated in mice. Finally, non-animal models involving human-derived cells shed light on specific effects of bacteria on human epithelial and immune responses. When considered alongside clinical cohort studies, experimental model systems have provided crucial evidence for the link between the neonatal gut microbiota and allergic disease, immensely supporting the stewardship of antibiotic administration in infants. The following review aims to describe the range of experimental models used for studying factors that affect the relationship between the gut microbiota and allergic disease and summarize key findings that have come from research in animal and *in vitro* models.

## Introduction

Experimental models enable the characterization of complex host responses ranging from cellular to systemic, contributing vitally to major advancements in immunology. Mice have long been the model of choice when studying host immunity and infection responses. The murine genome overlaps substantially with that of humans, and many key immune pathways are conserved. However, human and mouse immune systems are not identical, and findings in mouse models do not always translate directly to human application (1, 2). Non-animal models using cultured intestinal epithelial and immune cells, or miniature organ-like structures, have also been developed to determine specific effects of bacteria on various cell types (3, 4). Although this enables the study of cells derived specifically from humans, the response of cells grown in culture does not always reflect what occurs in the complex environment of a mammalian host. However, when used in conjunction with clinical human studies, both animal and *in vitro* models are critical to defining the mechanisms of host-microbiota interactions that affect immune development and atopic disease.

## Mouse models for studying the gut microbiota

Mouse models enable the manipulation of a microbial community growing within the complex environment of a mammalian host. Standard laboratory mice are considered “specific-pathogen free” or SPF, because they have tested negative for a set of disease-causing pathogens. These mice are raised in clean laboratory conditions and have a microbiota that is lower in diversity than that of humans, but complex enough to simulate a stable gut community with some colonization resistance capacity (5). Microbiota composition differs depending on the mouse vendor and housing conditions, which can lead to differences in baseline immunity and response to treatments (6). Additionally, it can be difficult to tease apart direct effects of microbes on the host from indirect effects that act through the existing microbiota when studying SPF mice. Pretreatment with antibiotics is often required to promote stable colonization with a newly introduced microbe, which can confound results. Despite these caveats, SPF mice are used extensively to demonstrate the effects of antibiotics and microbiota alterations on immune development and disease (7–11).

**Figure 1:**
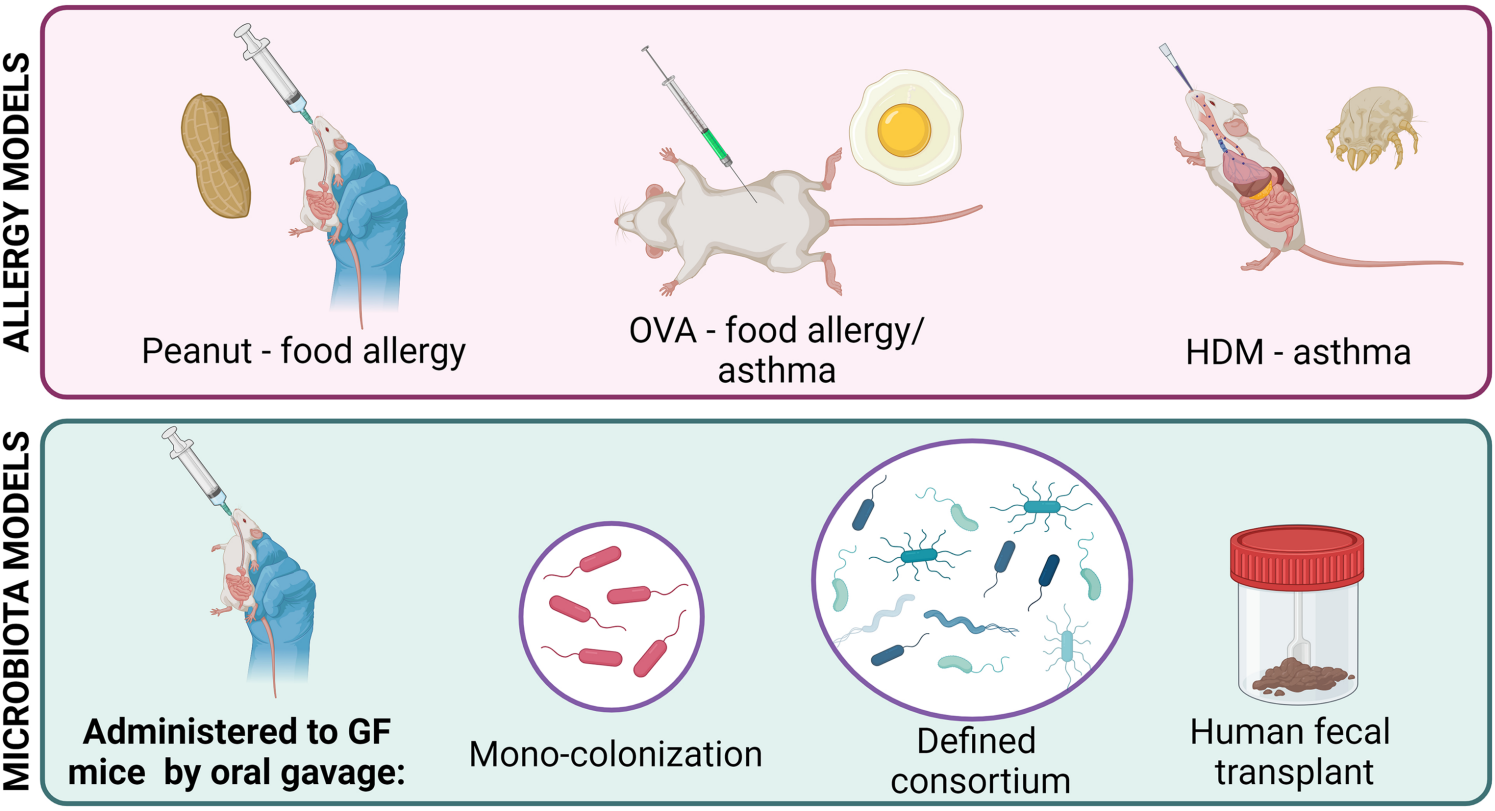
Common murine models for studying allergic disease and the gut microbiota.

As the microbiome field has grown, a variety of animal models have been developed to combat the issues faced with SPF mice (Figure 1). Germ-free (GF) mice, which are completely sterile, do not have a microbiota and can be more easily colonized than SPF mice (12). Mono-colonization of germ-free mice is commonly used to study the effects of one bacterial species at a time, independent of any confounding effects of other microbes in the environment. GF mice can also be colonized with a defined consortia of microbes to study simplified, specific communities and microbe-microbe interactions within the gut environment (5, 13, 14). Humanized mice, which are created by transferring human feces to GF mice, display a microbiota that is more diverse and complex than that of SPF mice and more similar to that of humans, facilitating the study of clinically relevant human-colonizing microbes *in vivo* (15, 16). However, some microbes found in humans require a host-specific niche and fail to colonize mice, limiting the utility of humanized mice (17). Lastly, transient colonization of mice with microbes that decline and disappear demonstrate the lasting effects of microbial exposure at specific developmental stages (18).

## Mouse models of allergic disease

Several mouse models have been developed to study the atopic immune response, with allergic asthma and food allergy being most common (Figure 1). Since all atopic diseases involve the same key pathways (Th2 cell activation, IgE production, mast cell degranulation, basophil hyperplasia), the basic process of allergy induction is similar between models: mice are first sensitized and then challenged with an allergen to initiate and then stimulate a Th2-mediated response (19–21).

There are two popular models of allergic asthma. The OVA model involves intraperitoneal injection, followed by intratracheal or intranasal administration, of ovalbumin derived from chicken egg (22). The house dust mite (HDM) model involves a series of intranasal exposures to protein from a common HDM species such as *Dermatophagoides pteronyssinus* (23). Both models stimulate a Th2-mediated response and lung histopathology (22, 24).

Food allergy models are difficult to develop because oral exposure to even concentrated allergenic food proteins results in oral tolerance and fails to induce a response (25). For oral induction, food allergens must be administered with adjuvants to induce a Th2-mediated response (26). For example, concentrated peanut extract can be administered orally with cholera toxin, resulting in elevated IgE production and anaphylaxis (27). Peanut allergens can also be administered epicutaneously to mice to induce Th2-mediated responses which can be further stimulated orally in the absence of an adjuvant, which may be explained by the link between skin barrier function and food allergy (28–30). Although peanut proteins show the highest allergenicity in mice, egg and milk allergies have been induced using adjuvants as well (26).

There are also models of atopic dermatitis and allergic rhinitis, the common forms of which also involve sensitization and challenge with ovalbumin, but are less common (31, 32). In humans, atopic dermatitis often precedes the development of food allergy and asthma in a sequential pattern termed the “atopic march” (33). The atopic march has been demonstrated in the NC/Nga mouse, commonly used to study atopic dermatitis (34). Epicutaneous OVA or peanut protein sensitization can also induce allergic manifestations at other sites (35). Our understanding of the microbiota's role in the atopic march is lacking, and murine models that display multiple allergies initiated via the skin should be utilized to further investigate factors that affect the progression of disease.

Studies of the microbiota and antibiotics using allergy models will be highlighted below.

## Atopic disease—insights from germ-free mice

GF mice, which lack a microbiota entirely, display a dramatically altered immune system (12). Intestinal and systemic immune compartments are largely underdeveloped or missing in GF mice. While some of these phenotypes can be rescued by colonization of germ-free mice at any age, others require colonization within the critical early-life window.

As in newborns, the immune systems of GF mice are skewed toward Th2 responses. Th1 and Th2 cells reciprocally regulate each other, and in the absence of microbial stimulation of Th1 pathways, Th2 responses go uncontrolled (36). This phenomenon supports the hygiene hypothesis, which posits that increased Th2 responses can be attributed to reduced microbial exposure and Th1 stimulation in modern, more hygienic environments (37, 38). Although mono-colonization of germ-free mice with certain commensals can reduce Th2 skewing, full restoration of normal T cell proportions requires colonization with a diverse microbiota (39).

Germ-free mice also display increased invariant natural killer T (iNKT) cells in the lung and colon. iNKT cells are found in higher proportions in individuals with severe and uncontrolled asthma (40). Concordantly, germ-free mice display increased iNKT-mediated inflammation in response to asthma models (41). Exposure to a specific antigen produced by the common commensal *Bacteroides fragilis* within the first two weeks of life is sufficient to rescue iNKT cell levels (42). iNKT cell levels cannot be restored in adult mice, implicating the preweaning period as an important moment in immune imprinting by the colonizing microbiota (12).

Serum IgE titers are also elevated in germ-free mice (43). This contributes to more severe anaphylaxis in food allergy models, and can only be rescued by colonization in the first few weeks of life (12, 43). Regulatory T cell development also requires antigenic exposure specifically during the early life period (44, 45). Many studies have demonstrated that these immunological features make GF mice more susceptible to asthma models (46–48). However, others have shown that certain aspects of the GF mouse allergy response are dampened (49), and that colonization of certain species can worsen allergy symptoms of germ-free mice (50). Together, studies in germ-free mice have shed light on the numerous pathways by which microbes in the gut can protect against or contribute to asthma and allergy development.

## The weaning reaction in mice

While colonization of GF mice at any age can rescue some of their immune alterations, exposure to microbes during the early-life window is required for complete restoration of proper immune function. Recently, this window has been well-defined in mice to be the first 3 weeks of life. At weaning, mice undergo a dramatic immune reaction to the influx of microbes and loss of passive immune factors in milk, which imprints their immune systems and susceptibility to disease for life (51). Although it is not clear whether the “weaning reaction” occurs in humans, the associations of breastfeeding and microbiota maturation in infancy with immune health later in life suggest that the human weaning period is also critical for microbiota and immune development (52, 53).

At weaning, the mouse microbiota diversifies and expands, displaying a bloom in Clostridia and Bacteroides which replace gamma-Proteobacteria and Lactobacillus species. This is similar to the microbiota shifts observed in humans from birth until 6 months of age (21, 54, 55). Milk contains several anti-inflammatory molecules, which gradually decline in concentration over time post-gestation. Epidermal growth factor (EGF) in milk delays the opening of Goblet-cell-associated antigen passages (GAPs) in the gut. As EGF levels decrease over the course of the first few weeks of life, GAPs open and allow for microbes to interact with and stimulate the immune system. In response to the developing microbiota and mucosa, GAPs close shortly after weaning. Antigenic exposure through GAPs drives regulatory T cell development, promoting tolerance of the gut microbiota and imprinting the immune system. This process is unique to the early-life period and cannot be induced in adult mice. Importantly, the weaning reaction protects against allergic inflammation later in life, and antibiotic exposure before but not after weaning significantly disrupts immune programming and contributes to worsened health outcomes (51).

The weaning reaction has not been demonstrated in humans, and while the exact mechanism may not be the same, the weaning reaction theory provides a potential explanation for the relationship between neonatal microbiota disruption and adverse health outcomes that is well-observed in humans. Accessing human neonatal tissue and blood to define a human weaning reaction would be immensely difficult. However, the deep characterization of this response in mice along with ample evidence of long-term effects of early-life antibiotics in infants provide clues to the process of microbe-mediated immune imprinting that occurs in humans.

## Antibiotics, the microbiota, and mouse models of asthma

Prompted by associations observed in human cohorts, the relationship between the gut microbiota and asthma has been studied and characterized extensively through colonization of GF mice or supplementation of SPF mice with different combinations of bacteria.

Numerous animal studies have identified specific immunomodulatory bacteria that protect against allergies. As mentioned above, colonization of germ-free mice with allergic infants is sufficient to increase anaphylaxis susceptibility (56). Through sequencing and host gene expression analysis, this effect was explained by protective effects of the Lachnospiraceae species, *Anaerostipes caccae*. Mono-colonization experiments confirmed that this species alone, which is elevated in healthy infants, contributes to oral tolerance and protects against anaphylactic responses. In another study, four genera of bacteria inversely associated with allergies in human infants were administered to mice and shown to ameliorate OVA-induced asthma (57). This was linked to the effects of specific bacterial metabolites on immune development.

Species of Bifidobacteria and Lactobacillus, which are known to be promoted by breastmilk and are inversely associated with allergies, have also been tested in animal models (58–60). Supplementation with *Bifidobacterium longum* and *Bifidobacterium breve* have been shown numerous times to limit allergic responses to various models by inducing regulatory T cells and dampening Th2 responses (48, 61–63). Lactobacillus species have also been shown to improve intestinal barrier integrity and promote Th1 responses, which limit allergic phenotypes (64, 65).

In all of these studies, bacteria known to be associated with health in humans were investigated and causally linked to allergy protection. Animal models have been key to moving from correlation to causation in our understanding of the multiple roles of the microbiota in immune development and have helped identify the specific bacteria and pathways that should not be disrupted during the neonatal period. In addition to providing insight into protective and beneficial bacteria, animal studies have demonstrated the effects of early-life antibiotic exposure on asthma.

Vancomycin treatment during neonatal but not adult life increases susceptibility to OVA-induced asthma in mice (8). This is linked to alterations in gut microbiota composition and diversity, and reduced colonic Treg cell levels. Both of these phenotypes were more drastic in neonatal than adult vancomycin-treated mice, and the period between birth and weaning was identified as the window during which antibiotic induced dysbiosis was found to significantly affect adult asthma outcomes (66). Similarly, Azithromycin treatment in early life increased IgE and Th2 responses to HDM-induced asthma (67). Interestingly, in this study, transfer of the azithromycin perturbed microbiota to adult germ-free mice did not transfer the phenotype. However, the offspring of these mice displayed worsened asthma outcomes, indicating that the effects of microbiota alterations on immune development must occur in early life. Dysbiosis induced by a combination of antibiotics has also been shown to impair oral tolerance by disrupting dendritic cell development in the gut (68).

Exposure to a single course of macrolide antibiotics at a clinically relevant dose during neonatal life is sufficient to reduce microbial diversity and shift community composition, which persists into adulthood. This was accompanied by permanent dampening effects on local and systemic immunity (69). In contrast, adult mice treated with the same antibiotic course displayed rapid microbiota recovery and did not show immune aberrations. Microbiota transfer from antibiotic-treated mice to germ-free mice conferred the altered immune phenotype in this study, demonstrating that the perturbed microbiota is sufficient to drive immune alterations associated with antibiotic exposure. Lynn et al. treated mice with ampicillin and neomycin until weaning and then aged them to 700 days (70). They found that early-life antibiotic exposure affects immune status, longevity, and metabolism even long after antibiotic exposure.

The studies highlighted above demonstrate the multitude of detrimental effects that antibiotic exposure in early life have on long-term health. Mouse studies have been vital in our understanding of both the specific effects of “protective” microbes and antibiotics on different facets of immune development, and the uniqueness of the infancy period in these processes. They provide strong support for limiting antibiotic administration in infants whenever possible.

## Non-animal models for studying antibiotics and allergic asthma

In addition to animal models, cultured human cells are frequently used to investigate the microbiota and allergic responses. Epithelial responses to microbial products can by studied by monoculture of human intestinal or lung epithelial cells. For example, Bifidobacteria species grown on human milk oligosaccharides induce anti-inflammatory responses in human-derived intestinal cells (71). Some commensal species and their metabolites have also affect barrier integrity of intestinal cell monolayers (72–75), either promoting or impairing barrier function, which has implications for allergy and asthma susceptibility (76, 77). However, allergic disease involves communication between the epithelium, the mucosal immune system, and systemic circulation, and it is difficult to define the mechanisms and effects of microbe-immune crosstalk using only one cell type. Co-culture systems involving epithelial cells grown with dendritic cells, macrophages, and lymphocytes have been developed to combat this issue.

In one study, peripheral blood mononuclear cells (PBMCs) were isolated from atopic or non-atopic individuals and co-cultured with epithelial cells exposed to microbial antigenic stimulation to demonstrate that commensal microbe exposure limits the allergic response (78). Co-culture of PBMCs with intestinal epithelial cells has also been used to mechanistically link Bifidobacteria and other commensal species to regulatory T cell responses (79, 80). In another study, human-derived naïve T cells were exposed to fecal water from infants that received *Bifidobacterium infantis* supplementation or fecal water from control infants. This enabled the identification of a specific microbial metabolite produced by B. *infantis* in the infant gut that skews T cell phenotypes away from allergic (Th2 and Th17) and towards Th1 phenotypes (81). More recently, co-culture systems involving many cell types have been developed. Zuurveld et al. designed a model involving epithelial cells, dendritic cells, T and B cells, and mast cells and were able to simulate the entire allergic pathway from epithelial cell allergen exposure to mast cell degranulation and IgE production *in vitro* (4).

Finally, in addition to mono- and co-culture systems, 3D structures that better simulate organ-level biology can model microbe-immune communication. Organoids and gut-on-a-chip devices are derived from human intestinal stem cells and morphologically mimic 3D intestinal tissue, displaying crypts, villi, Paneth cells, mucous production, and distinct apical and basolateral compartments (3, 82). These models are only beginning to be used to study microbe-host interactions that influence systemic health but offer a promising physiologically relevant alternative to animal models (83, 84).

## Conclusions

Experimental models have characterized the complex and dynamic processes of microbe-mediated immune development that occurs in early-life and validated key taxa that drive protective and beneficial immune processes. They have provided ample evidence that antibiotics have detrimental effects on gut microbiota composition, long-term health, and allergy in a controlled setting. They have also defined the features of immunologic development that occur during the critical early-life window, and illustrated the imprinting effects of the microbiota during this period. While lab rodents and cultured cells do not replicate the human gut and immune system identically, the findings highlighted above strengthen and complement conclusions from human association studies, drawing clear mechanistic links between antibiotic exposure, reduced microbiota diversity, and allergic outcomes.
